# The partial merger of two dolphin societies

**DOI:** 10.1098/rsos.211963

**Published:** 2022-08-03

**Authors:** Nicole Danaher-Garcia, Richard Connor, Gavin Fay, Kelly Melillo-Sweeting, Kathleen M. Dudzinski

**Affiliations:** ^1^ Department of Biological Sciences, Institute of the Environment, Florida International University, 3000 NE 151st Street, North Miami, FL 33181, USA; ^2^ Dolphin Communication Project, PO Box 7485, Port Saint Lucie, FL 34984, USA; ^3^ University of Massachusetts Dartmouth, 285 Old Westport Road, North Dartmouth, MA, USA; ^4^ Dolphin Alliance Project, PO Box 6449, New Bedford, MA 02742, USA; ^5^ School for Marine Science and Technology, University of Massachusetts Dartmouth, 836 S Rodney French Boulevard, New Bedford, MA 02744, USA

**Keywords:** intergroup interactions, affiliative interactions, Atlantic spotted dolphins, *Stenella frontalis*, group mixing, immigration

## Abstract

Interactions between mammalian social groups are generally antagonistic as individuals in groups cooperate to defend resources from non-members. Members of the family Delphinidae inhabit a three-dimensional habitat where resource defence is usually impractical. Here, we describe a long-term partial fusion of two communities of Atlantic spotted dolphins (*Stenella frontalis*). The northern community, studied for 30 years, immigrated 160 km to the range of the southern community, observed for 20 years. Both communities featured fission–fusion grouping patterns, strongest associations between adult males, and frequent affiliative contact between individuals. For the 5-year period following the immigration, we found members of all age classes and both sexes in mixed groups, but there was a strong bias toward finding immigrant males in mixed groups. Some association levels between males, and males and females, from different communities were as high as the highest within-community associations. Affiliative contacts indicate that these individuals were forming social relationships. The mixing of two separate social groups with new bond formation is rare in terrestrial mammal groups. Such mixing between spotted dolphin groups suggests that adaptations to respond aggressively to ‘outsiders’ are diminished in this species and possibly other ecologically similar dolphins.

## 1. Introduction

Many birds and mammals live in social groups whose interactions with outsiders are characterized by animosity. Group members jointly defend space containing resources, including mates, against others [1] in areas of home range overlap or delimited by territorial boundaries [2]. Members of one sex, typically females in mammals, stay in their natal area or group while maturing members of the other sex disperse [3]. Males may move into bachelor groups (e.g. golden snub-nosed monkeys, *Rhinopithecus roxellana* [4]) or directly into non-natal groups. In some species (e.g. rhesus macaques, *Macaca mulatta* [5]), individual males preferentially move into groups that include male kin, while in others, groups of males may disperse together to form coalitions and take control of non-natal groups (e.g. African lions, *Panthera leo* [6]). Chimpanzees (*Pan troglodytes*) provide an example where the pattern is reversed; males are philopatric, cooperating with other resident males to defend a feeding territory and resident females that benefit from it, while females disperse into non-natal groups [3,7].

Members of the dolphin family (Delphinidae) are large-brained, gregarious mammals with a range of social structures, from stable matrilineal groups that fit the traditional definition of ‘multi-level societies' (e.g. killer whales, *Orcinus orca* [8]) to those such as bottlenose dolphins (*Tursiops* sp.) whose ‘fission–fusion’ grouping pattern resembles that of chimpanzees and spider monkeys (*Ateles paniscus* [9]). In these societies, we find evidence of complex social relationships maintained with affiliative (i.e. ‘friendly’) contact behaviours (much like primate grooming) and synchrony [10–13].

Patterns of philopatry and dispersal are not well understood for many cetaceans, but bisexual philopatry, rare in terrestrial mammals, appears common for cetacean species (e.g. resident killer whales [8]; bottlenose dolphins [9]; long-finned pilot whales, *Globicephala melas* [14]). Bisexual philopatry may take the form of geographical philopatry, in which individuals maintain their natal range in their adult home range (e.g. bottlenose dolphins [9,15]), or social philopatry, in which individuals remain in their natal area and within their natal social group (e.g. resident killer whales [8]). The relatively low cost of locomotion, along with large ranges, brings individuals into contact with many others, including unrelated potential mates [16]. In Shark Bay, Western Australia, a chimpanzee-like fission–fusion grouping pattern with complex male alliances is not matched with a primate-like closed group structure; rather, these dolphins live in an open social network comprising hundreds of individuals with a mosaic of overlapping male and female home ranges [17].

**Figure 1.**
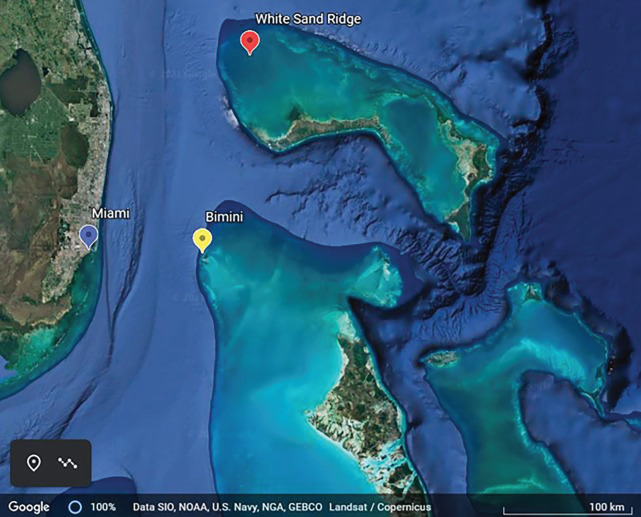
Map of The Bahamas in relation to southern Florida, USA, focused on the Little Bahama Bank and the northern Great Bahama Bank. The yellow marker indicates the Bimini islands that are approximately 80 km east of Miami (blue marker). The red marker shows the approximate location known as the WSR on the Little Bahama Bank, approximately 160 km north of the northernmost edge of the Great Bahama Bank. Map credit: Google Earth.

A fission–fusion grouping pattern is often described in other species of in- or near-shore delphinids (e.g. Australian humpback dolphins, *Sousa sahulensis* [18]; white-beaked dolphins, *Lagenorhynchus albirostris* [19]), including Atlantic spotted dolphins (*Stenella frontalis*) in The Bahamas, that have been the subject of two long-term studies [20,21]. Our group, the Dolphin Communication Project (DCP), has been studying a resident group of approximately 120 spotted dolphins in an area of roughly 250 km^2^ on the northwestern portion of the Great Bahama Bank in the coastal waters around Bimini, The Bahamas, since 2003 [20]. Another research group has studied resident spotted dolphins on the White Sand Ridge (WSR), a 480 km^2^ area on the Little Bahama Bank, north of Grand Bahama Island since the mid-1980s [21]. The WSR community included approximately 100 individuals before dropping by a third following two hurricanes, but had recovered to 85 individuals by 2012, the year before the immigration event described here [22]. WSR lies about 160 km north of the northernmost range of the Bimini group. The two study sites on the Little Bahama Bank and Great Bahama Bank are shallow (WSR 6–16 m [21]; Bimini 6–12 m [20]) but separated by a deep water channel (up to 2000 m; see figure 1).

Long-term studies in Bimini and WSR demonstrated that both dolphin societies showed strong site fidelity, year-round residency, and shared social structure characteristics including group size, fission–fusion dynamics and long-term, typically same sex, preferential associations [20,21,23]. The WSR dolphins were found to live in three social clusters delineated by higher levels of association within than between clusters and differential use of habitat [23]. No clustering was observed in the Bimini population [20]. The WSR dolphins also form two levels of male associations featuring long-term stable pairs or trios and secondary groupings [21]. While there were high association levels among some males in Bimini, the two levels of male association reported in WSR were not in evidence [20]. Spotted dolphins in The Bahamas engage in frequent affiliative contact behaviour [24–28], even with sympatric bottlenose dolphins (*Tursiops truncatus*) [29]. There is no evidence that members of either spotted dolphin society defend territories or engage in resource defence against other groups, as so commonly occurs in terrestrial mammal societies.

The bisexual philopatry characteristic of many delphinids is also found in both the WSR and Bimini spotted dolphin communities. Elliser & Herzing [23] state that WSR individuals of both sexes remain in the same area or community through adulthood. The Bimini dolphins also appear to conform to a model of bisexual philopatry, as males and females first observed as dependent calves and young juveniles have remained in the community into adulthood (DCP, unpublished data, 2001–2020). However, we cannot exclude some emigration since we cannot determine if individuals that vanish have died or emigrated. It is unclear whether the 0–2 non-calf individuals added to the WSR and Bimini catalogues each year emigrated from outside the area or were, for example, previously spotless unidentified calves that had developed spots and so become identifiable (WSR: [21]; Bimini: DCP, unpublished data, 2001–2020).

In the 10 years prior to 2013, no interaction between dolphins from the two areas was observed [20,21,23,30]. In 2013, 52 individuals from WSR immigrated south to the Great Bahama Bank, including the Bimini study site. The immigrants included all sex and age classes: 45 dolphins from one WSR cluster and 7 from the other two, as follows: 25 males (15 adults, 5 juveniles and 5 calves) and 27 females (16 adults, 8 juveniles and 3 calves) [22]. Also beginning in 2013, DCP documented the occurrence of 46 immigrants from WSR (DCP, unpublished data, 2013–2019), which represent the majority of the 52 emigrants described by Herzing *et al.* [22].

From 2013 through 2018, many WSR emigrants were observed associating with Bimini dolphins. Given the generally closed nature of terrestrial mammal societies, these observations were surprising. Cases where dolphin societies violate terrestrial ‘rules’, such as bisexual philopatry in killer whales and the open social network of Indo-Pacific bottlenose dolphins (*Tursiops aduncus*) in Shark Bay, broaden our understanding of social evolution in the terrestrial and marine spheres. The immigration of a large group of WSR spotted dolphins, including all age and sex classes, into the Bimini area, and their subsequent association with Bimini dolphins, was also unexpected. We therefore conducted a detailed study of sex- and age-specific associations between Bimini and WSR dolphins, using our previous study on Bimini dolphins as a baseline [20]. We predicted that, if associations between individuals from the two previously separate societies reflected the development of new social bonds, we would observe affiliative tactile interactions between WSR and Bimini individuals. What emerges is an unprecedented partial merger of these two previously unassociated and distant social networks of Atlantic spotted dolphins.

## 2. Methods

### 2.1. Study area and species

Data were collected west and north of the islands of Bimini, The Bahamas, in the northwest portion of the Great Bahama Bank (figure 1; for further details related to this study site, see [31]). DCP researchers have been studying the spotted dolphins around Bimini since 2001, with systematically collected data available from 2003 to 2020. Spotted dolphins are classified by age based on the gradual development of spot pigmentation on their bodies [32,33]. Calves are born grey-white without markings and begin developing black spots primarily along the ventral and lateral sides at approximately 3–4 years; once independent and ‘speckled’ with dark spots, they are juveniles [33]. Spot development continues, mainly with dark ventral spots and light dorsal spots; when this spot development is extensive, they are considered ‘mottled’ and have reached the young/subadult phase [33]. Adults are classified by a ‘fused’ pattern of spots [33]. A dolphin's unique spot pattern is also used along with permanent scars to identify individuals. Sex is ascertained through observation of the genital area including erections (males) and recurrent associations with calves (females) or lack of such associations (males). Spotted dolphins in this area are habituated to boats and human swimmers; however, they are not provisioned and physical contact with humans is discouraged.

Since 2004, most additions to the DCP spotted dolphin identification catalogue were from births, with calves or young juveniles added once they were observed with a permanent scar or distinctive spot pigments. Between 2003 and 2012, the addition of older dolphins to the catalogue was infrequent and generally a result of poor photo/video quality from first sightings or initial sightings lacking photographic or video documentation. Beginning in 2013, there was a large addition of subadult/adult dolphins into DCP's catalogue. This influx of older dolphins that could not be matched to catalogued or temporary IDs suggested that these new dolphins had emigrated from elsewhere.

We compared images of new dolphins seen around Bimini since 2013 to images of spotted dolphins seen previously on WSR. Forty-six spotted dolphins (23 females: 10 adults, 9 subadults, 3 juveniles, 1 calf; 22 males: 12 adults, 6 subadults, 3 juveniles, 1 calf; 1 unknown sex: 1 adult) newly observed around Bimini were photographically matched with individuals observed on WSR prior to 2013 (DCP, unpublished data, 2016–2020) and are hereafter referred to as WSR dolphins/individuals for simplicity. Including the WSR individuals, there were 163 individual spotted dolphins in the DCP Bimini spotted dolphin catalogue (DCP, unpublished data, 2003–2018); in 2018, approximately 5% were calves, 25% juveniles, 20% young adults and 50% adults. There was a roughly 1.3 : 1 female to male ratio; however, sex was unknown for approximately 17% of catalogued individuals.

### 2.2. Data collection

DCP collaborates with local ecotour companies that generally operate boat trips in the 4–5 h before sunset; as such, data collection for this study most often occurred during this time window. Data were collected between late April and early September each year with occasional effort outside these months. Surveys were conducted in all weather conditions except severe wind or storms (Beaufort greater than 5, intense thunderstorms, or severe rain) with the majority of surveys occurring in Beaufort less than 4. Sightings were periods of time with dolphins in view from the boat while encounters were defined as underwater observations of 3 min or more [34]. Encounters occurred while the boat was in motion or drifting.

Cameras in waterproof housings were used to collect video data during encounters (protocols outlined in [26,35,36]) using a focal-follow, all-occurrence sampling protocol [37,38] as outlined in Dudzinski [34,39] and Melillo *et al.* [31]. Still images were collected using underwater point-and-shoot cameras following an *ad libitum* sampling method [37] to opportunistically collect images of as many dolphins from as many angles as possible to record overall group composition.

Tactile behaviour was recorded from underwater video data (2013–2018) following an all-occurrence event sampling protocol [37]. Identities of initiating and receiving dolphins, with each dolphin's age, sex and origin (i.e. Bimini or WSR), were documented. A dolphin was defined as ‘initiator’ if its movement caused contact with a receiver. The receiver's response to contact (i.e. positive, neutral or negative) was also logged, if discernable. The receiver's response was considered positive when the dolphin responded with affiliative behaviour, neutral when there was no visible response, and negative when the receiver left or responded with aggressive behaviour (see [40] for details).

### 2.3. Data analyses

Only sightings involving an underwater encounter were included in this study and only individual dolphin identities confirmed through photographs or video were used in analyses. The integration of Bimini and WSR dolphins was analysed in R, v. 3.5.2 [41], the *car* [42] and *multcomp* [43] packages. The yearly change in the proportion of mixed groups (composed of Bimini and WSR individuals) observed from 2013 to 2018 was investigated using a generalized linear model (GLM) with a binomial distribution and year as a factor, a type III ANOVA and Tukey's HSD *post hoc* test.

Analysis of dyadic associations was conducted using SOCPROG 2.9 [44]. Coefficients of association (COAs) were determined using the half-weight index (HWI; see [45] for definition). The HWI is appropriate when there is incomplete identification of group membership, as in this study [45,46].

Because there was only one mixed group in 2013, subsequent analyses of association were limited to 2014–2018. For association, interaction and network analyses, data were restricted to individuals sighted at least twice per year or 10 times in 2007–2012 (following [20]) and four times in 2014–2018. A lower number of sightings was allowed for the period after the arrival of WSR dolphins in an effort to include more WSR females, which were sighted significantly less than WSR males (*t*-test: *t* = 3.23, *p* = 0.002; electronic supplementary material, table S1). Calves were not included under the assumption that their associates would be dependent upon their mother's associations.

To test for preferred or avoided associations, randomized permutations were set to 5000 with 1000 trials per permutation, the number of permutations and trials at which *p*-values stabilized for all years and pooled periods [46]. All analyses were completed using ‘permute associations within samples' because it accounts for differences in gregariousness among individuals. It is also the most robust option in SOCPROG and is appropriate for an open study population in which individuals move in and out of the area due to emigration, birth or death [44]. Network graphs (sociograms) representing associations between individuals in the population were created using UCINET 6 for Windows [47].

Clustering in the network is defined by subgroups with higher COAs among individuals in the same group and lower COAs among individuals in different groups. We examined clustering in this study using hierarchical agglomerative cluster analysis [46]. The average-linkage method was used to produce a dendrogram in which individuals lie along one axis and their degree of association lies along the other [46]. Since dendrograms can be overinterpreted, the cophenetic correlation coefficient (CCC; range from 0 to 1) was calculated to determine how well the resulting dendrogram corresponded to actual association rates; CCC greater than 0.8 indicates an acceptable representation [44,46]. Additionally, to assess the value of the resulting clustering scheme, modularity, defined as the difference between the proportion of total association within clusters and the expected proportion, was measured using an eigenvector-based method [46]. A modularity greater than 0.3 suggests notable division of the population into clusters [48]. Finally, clustering was tested using non-metric multi-dimensional scaling in which a reduced dimensional representation of dyadic associations is created [46]. Dyads that are more strongly associated are plotted more closely together than less associated dyads [44]. The number of dimensions presented in the plot is increased until stress no longer decreases; stress less than 0.20 suggests a useful display [44]. The starting configuration was set to ‘random’ and the analysis was conducted three times to confirm that the final representation was optimal.

Underwater contact exchanges were investigated in R. To reduce autocorrelation, only the first contact per day per identified dolphin, as initiator and as receiver were used. Chi-square analysis was used to assess the influence of age, sex and origin in partner preference and receiver response.

To evaluate if WSR males moved to Bimini to access more mating opportunities, we calculated the operational sex ratio (OSR) of WSR dolphins prior to the move and WSR and Bimini dolphins after. Following Herzing *et al.* [22], we calculated OSR using two numbers for males: the number of adult males only (which might be reproductively dominant) and the number of adult and subadult males (the number of males that are of reproductive age). The number of receptive females was the number of reproductively mature females minus those who were pregnant or in the first 2 years of lactation (given a 4-year interbirth interval and 1-year gestation [33]). As the number of reproductively available females will vary annually, we calculated an OSR range for the study period.

## 3. Results

### 3.1. Sightings and group structure

From 2007 to 2012, 437 dolphin surveys were conducted, including 325 trips with spotted dolphin sightings and 423 encounters (see [20]). In 282 dolphin surveys between 2013 and 2018, spotted dolphins were sighted on 248 trips with 402 encounters, of which 76 encounters included WSR dolphins (electronic supplementary material, table S2).

Forty-five Bimini dolphins were observed in groups with WSR dolphins including six adult females, nine adult males, six subadult females, four subadult males, 11 juvenile females and nine juvenile males (electronic supplementary material, table S1). Of the 46 WSR dolphins confirmed around Bimini, 27 were observed in groups with Bimini dolphins including six adult females, nine adult males, five subadult females, five subadult males, one juvenile female and one adult of unknown sex (electronic supplementary material, table S1). In sum, we sighted the majority of known immigrants from WSR [22] and observed the majority of Bimini dolphins with immigrants, but just over half of the WSR immigrants with Bimini dolphins.

**Figure 2.**
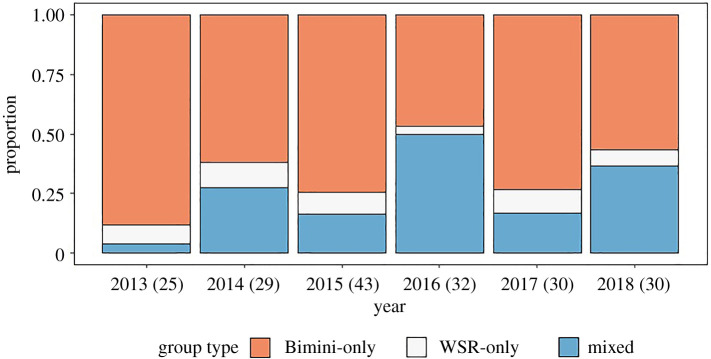
The proportion of Bimini-only, WSR-only and mixed-origin groups in each year after immigration of WSR dolphins (2013–2018). Total number of groups observed is shown in parentheses next to the respective year.

Mixed groups were observed throughout each field season except in 2013 when the only mixed group sighting occurred during the sixth survey of the season (of 39 surveys; electronic supplementary material, table S2). From 2013 to 2018, there was a significant increase in the proportion of mixed groups composed of both Bimini and WSR dolphins (ANOVA, *F(5)* = 5.47, *p* < 0.0001; figure 2); the proportion of mixed groups was significantly greater in all years as compared to 2013 (GLM, *p* < 0.05), except in 2017 (GLM, *p* = 0.144). Excluding 2013, there was still a difference in the number of mixed groups across years (ANOVA, *F(4)* = 4.04, *p* = 0.004); however, only 2016 appears to be significantly different from the other years (GLM, *p* = 0.02). Furthermore, 2016 is only significantly different from 2015 to 2017 (Tukey's HSD, *p* = 0.02), and there is no evidence of an increasing proportion of mixed groups over the whole period.

With the addition of the WSR immigrants, the number of dolphins in the Bimini study area increased from about 120 to over 160 individuals, but average group size did not change (one-tailed *t*-test, *p* = 0.52). In the 6 years before the WSR dolphins immigrated to Bimini (2007–2012), group size in Bimini ranged from two to 50 individuals ($\bar{x}$ = 10 ± 6.3, mode = 10). After the immigration event, of 197 groups (single- and mixed-origin) for which group size was recorded between 2013 and 2018, group size ranged from two to 75 dolphins ($\bar{x}$ = 10 ± 8.3, mode = 4). However, the size of Bimini-only groups was significantly smaller after the immigration of WSR dolphins (one-tailed *t*-test, *p* = 0.002); groups ranged in size from two to 26 individuals, with 75.2% of Bimini groups having 10 or fewer individuals ($\bar{x}$ = 8 ± 5.4, mode = 4). The size of WSR-only groups was similar (range = 2–12, $\bar{x}$ = 8 ± 4.7, mode = 4). By contrast, the size of mixed-origin groups ranged from three to 75 individuals ($\bar{x}$ = 15 ± 11.2, mode = 8; electronic supplementary material, table S3), which significantly differed from average group size of Bimini-only or WSR-only groups (ANOVA, *F(1)* = 12.6, *p* < 0.0001).

In mixed-origin groups, there were between one and 10 confirmed WSR dolphins. The proportion of WSR dolphins in mixed-origin groups ranged from 0.03 to 0.70 ($\bar{x}$ = 0.36, mode = 0.25; electronic supplementary material, table S3). On average, Bimini females made up the largest proportion of mixed groups, followed by Bimini males and WSR males; WSR females were occasionally present but in low numbers and never comprised more than 1/3 of group composition (electronic supplementary material, table S3). Of all possible sex and age class combinations, mixed age/sex groups (composed of four age classes and both sexes) were most frequently observed during observations of mixed-origin groups (94%).

### 3.2. Association rates and cluster analyses

**Figure 3.**
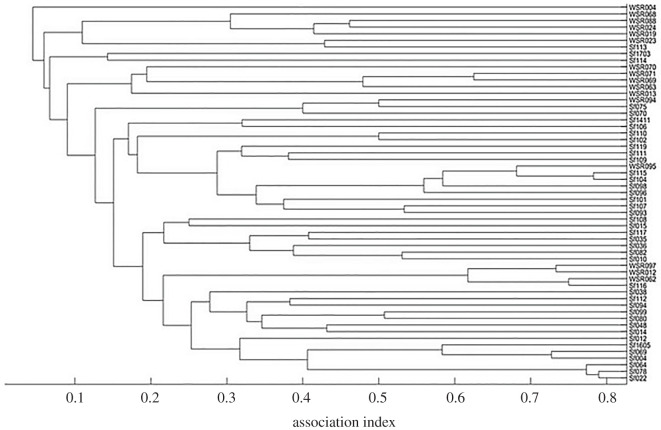
Hierarchical agglomerative cluster analysis (CCC = 0.77) of individuals included in analyses between 2014 and 2018. Pairs of individuals connected farther to the right had a stronger association throughout the study period. Association index value is given along the horizontal axis.

The cluster analysis and resulting dendrogram (figure 3) revealed no evidence that the Bimini and WSR dolphins associated in separate communities from 2014 to 2018. A non-metric multi-dimensional scaling analysis (electronic supplementary material, figure S1) also demonstrates considerable integration between Bimini and WSR dolphins. Maximum modularity of cluster analysis reached 0.133.

**Figure 4.**
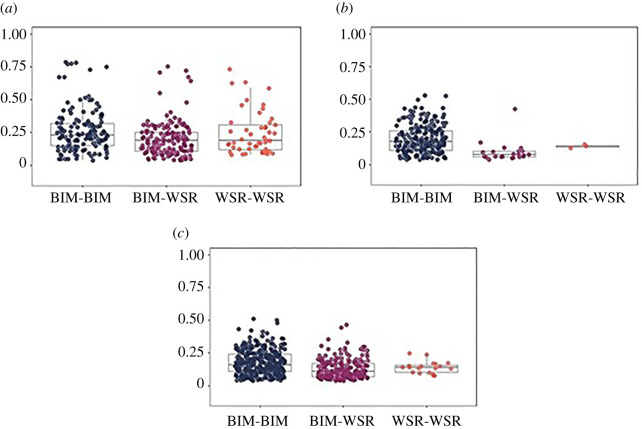
Boxplots of association indices (COAs) for 2014–2018 with actual values (coloured circles) for each dyad. The solid grey horizontal lines represent medians, the boxes represent quartiles and the vertical lines represent the 1.5 × interquartile ranges. (*a*) Male dyads, (*b*) female dyads and (*c*) male–female dyads.

The distribution of COAs for 2014–2018, by sex and origin, is shown in figure 4. Many more males (*N* = 13) than females (*N* = 3) from WSR met criteria for inclusion in COA analyses. The cluster of eight high COAs (range = 0.67–0.79) among Bimini males (figure 4*a*) involves five adult and five subadult males, including two adult pairs. Five mixed-origin male relationships fall in this same range (range = 0.64–0.75), including one Bimini adult, two WSR adults, two Bimini subadults and two WSR subadults. The only high mixed female–female COA (0.43) was between two juveniles (figure 4*b*). While there is no distinct cluster of high male–female COAs in any groups, there were 59 male–female pairs that exceeded the 75th percentile (COA = 0.17) for Bimini male–female associations (figure 4*c*). Mixed-origin male–female pairs included eight Bimini adult females, five Bimini subadult females, seven Bimini juvenile females, one Bimini adult male, one Bimini juvenile male, five WSR adult males, five WSR subadult males and two WSR subadult females. Among these associations were 16 pairings of Bimini adult females and WSR adult males. There were no high associations between WSR adult females and Bimini adult males, but only one adult WSR female met criteria for inclusion. Mixed-origin pairs with high association coefficients for the entire 2014–2018 study generally had similarly high association levels in the early versus late periods of the study (2014–2015 versus 2017–2018; electronic supplementary material, table S4).

**Figure 5.**
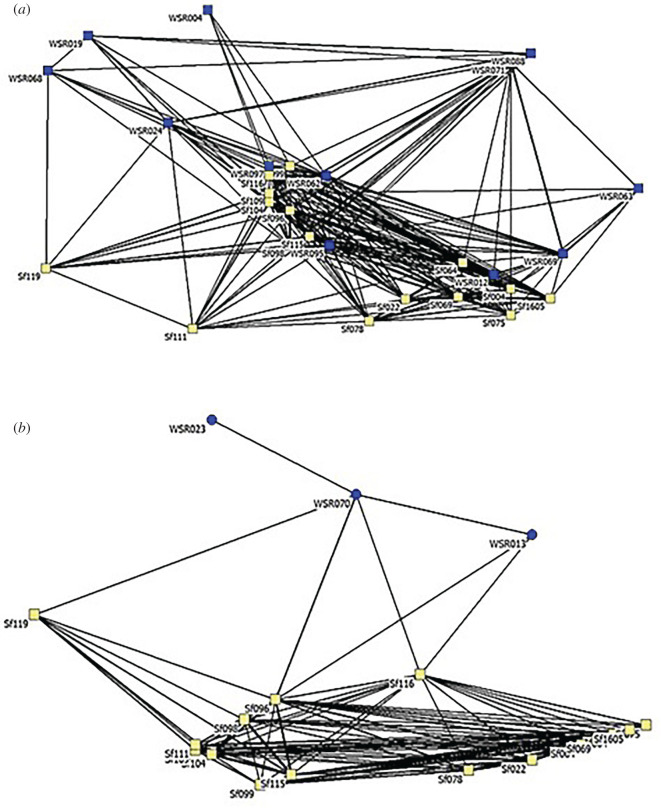
Sociogram representing association (2014–2018) between Bimini males (yellow) and WSR dolphins (blue): (*a*) WSR males and (*b*) WSR females. For all sociograms (*a*,*b*, and figure 6*a*,*b*), the following standards were used: only pairs with COA greater than the overall mean (*x̄* = 0.14) are displayed; line thickness is proportional to the strength of association; individuals are arranged using multi-dimensional scaling, in which dolphins with closer associates throughout the 6-year period are plotted more closely together and more centrally.

**Figure 6.**
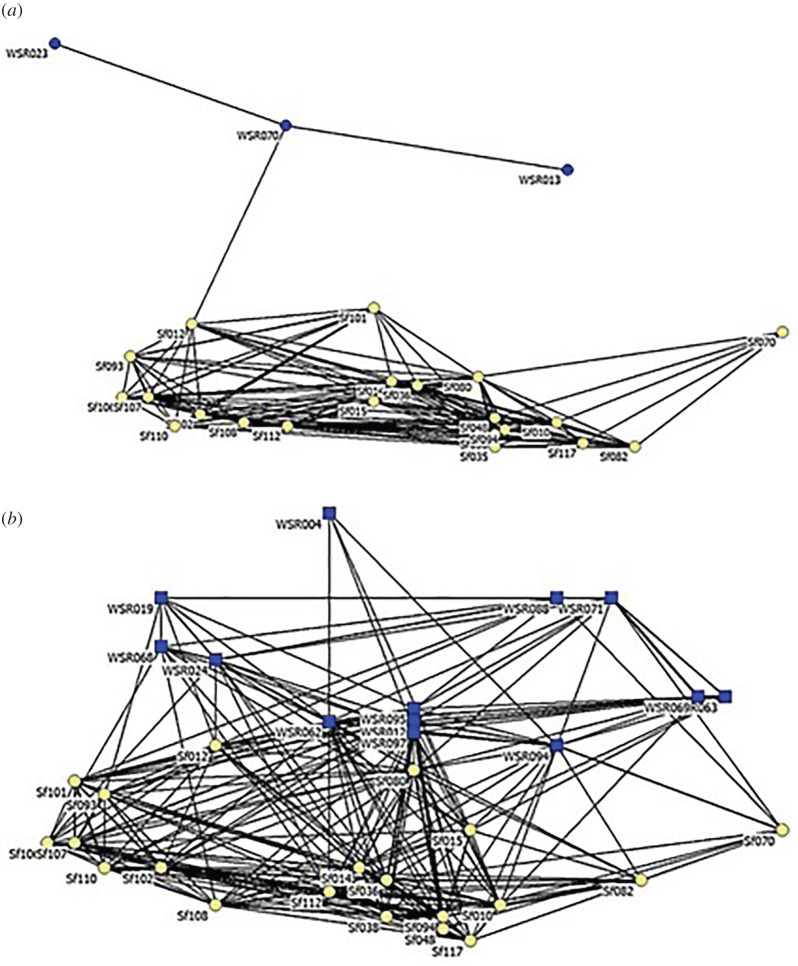
Sociogram representing association rates (2014–2018) between Bimini females (yellow) and WSR dolphins (blue): (*a*) WSR females and (*b*) WSR males.

Sociograms depicting associations between pairs divided by origin and sex (Bimini and WSR males: figure 5*a*; Bimini males and WSR females: figure 5*b*; Bimini and WSR females: figure 6*a*; Bimini females and WSR males: figure 6*b*) include pairs with COA greater than the overall mean (*x̄* = 0.14).

### 3.3. Analysis of interactions via contact events

A total of 655 contact events were recorded during 76 mixed group encounters after WSR dolphins immigrated to the Great Bahama Bank (2013–2018), with 394 events remaining after selecting the first contact per identified dolphin per day as initiator and receiver. Of these 394 contacts, 290 occurred between Bimini dolphins, 86 between Bimini and WSR dolphins, and 18 between WSR dolphins. The greater number of interactions between Bimini dolphins is likely in part due to the generally greater number of Bimini dolphins observed during encounters: on average, WSR dolphins made up approximately 1/3 of group composition (electronic supplementary material, table S3).

Affiliative interactions between WSR and Bimini dolphins were not limited to a particular age or sex class. Of 26 Bimini dolphins observed in WSR interactions, there were two adult females, five adult males, three subadult females, three subadult males, six juvenile females, five juvenile males and two juveniles of unknown sex. The 18 WSR dolphins included four adult females, six adult males, two subadult females, four subadult males, one juvenile female and one adult of unknown sex. Significantly more contacts occurred between individuals from the same origin (*X^2^* = 125.09, *p* < 0.0001). Specifically, Bimini dolphins initiated contact more often with other Bimini dolphins rather than WSR dolphins (*X^2^* = 9.62, *p* < 0.05).

Bimini juveniles initiated the most contacts (*X^2^* = 9.89, *p* < 0.05) while WSR dolphins initiated contact equally across age classes. Overall, juvenile dolphins initiated contact with other juveniles significantly more often than any other age combination (*X^2^* = 97.30, *p* < 0.0001). For WSR and mixed-origin dyads, more contacts were observed when dyads were of the same sex (WSR: *X^2^* = 5.56, *p* = 0.02; mixed: *X^2^* = 4.65, *p =* 0.03).

There were significantly more positive and neutral responses by a receiver to contact initiated by another dolphin than negative responses for Bimini-only and mixed-origin dyads (Bimini: *X^2^* = 110.45, *p* < 0.0001; mixed: *X^2^* = 22.42, *p* < 0.0001). Nine out of 11 negative responses recorded for mixed pairs occurred between Bimini females and WSR males; six of these contacts were initiated by the female. No negative responses were recorded for contact between WSR dolphins.

For mixed-origin adult/subadult male dyads, all pairs that had over twice the average COA were seen in physical contact at least once. Contacts were initiated equally by Bimini and WSR males, though specific males did initiate more contacts than others. By contrast, the Bimini–WSR female dyad with the highest COA was not observed making physical contact. Almost all contact recorded between Bimini and WSR females occurred between juveniles with COAs below the overall average. Contacts were initiated equally by females of both origins. All recorded contacts initiated by WSR females were directed towards juvenile Bimini females. However, all observed contact between WSR male and Bimini female adults occurred in dyads with COAs near the overall average. No contacts were observed between Bimini male adults and WSR female adults.

### 3.4. Operational sex ratio

The OSR for spotted dolphins around Bimini from 2007 to 2012 ranged annually from 0.75 to 1.4 including adults and possibly reproductive subadults, and 0.27–0.71 for adults only. Herzing *et al.* [22] defined OSR differently from the standard (i.e. the ratio of potentially receptive males to receptive females; e.g. [49]), so we recalculated OSR for WSR dolphins using the number of males and females provided in their study; prior to 2013, OSR on WSR was about 1.64 (0.64 adults only). Including the WSR individuals observed around Bimini after 2013, OSR for all spotted dolphins in this area ranged annually from 1.42 to 1.70 (0.92–1.1 adults only).

## 4. Discussion

We documented a long-term ‘partial’ fusion of two Atlantic spotted dolphin groups previously separated by 160 km and a deep channel, with no interaction previously observed. The first mixed-origin group was documented off Bimini in 2013, with mixed-origin groups regularly observed since then. Some dyads of mixed community male–male and male–female associations were consistently strong over the 5-year study and as strong as those among Bimini dolphins. Affiliative contacts suggest that associating WSR and Bimini individuals formed new social bonds. There was no evidence of a steady increase in the proportion of mixed group sightings during 2014–2018, but it remains possible that further integration will occur if subsequent generations grow up in the same area. Notably, intergroup aggression was not observed, as one might expect if dolphins lived in closed, defended social groups.

The strong bisexual philopatry in this population makes it very unlikely that the Bimini and WSR individuals that formed new social bonds were genetically related. Moreover, the histories of the individuals that formed new bonds are well known. All Bimini males that formed relatively strong bonds with immigrant WSR males were born in Bimini. This excludes the possibility that the Bimini males had previously immigrated from WSR and were joined by WSR relatives.

Such a full or partial fusion of previously separate social groups is very unusual in terrestrial mammals. In primates, for example, unusual numbers of individuals joining new groups, or long-term group fusions, are rare and usually the result of external pressures (e.g. predation or habitat loss) causing a decline in group size that favours group merger for survival. In vervet monkeys (*Cercopithecus aethiops*) in Amboseli National Park, Kenya, groups were forced to fuse when the number of adults declined to one or none [50]. The Amboseli population was already declining, with the number of adults significantly impacted by predation [51]. Similarly, group size in a wild Japanese macaque (*Macaca fuscata yakui*) troop was decreasing due to low birth rates and high infant mortality [52]. After the last male left the group, the remaining individuals fused with a neighbouring troop [52].

Bottlenose dolphins in Shark Bay do not live in a closed social group but instead have an open society with overlapping male and female home ranges [17,53,54]. We suggest that the spotted dolphin group fusion documented off Bimini is a different manifestation of the same phenomenon—a reduced importance of group social boundaries and the associated adaptations that trigger group-level aggression toward non-members. We are not suggesting that unfamiliar spotted dolphins would not show aggression, only that such a response is significantly muted in comparison to most social terrestrial mammals, allowing the dolphins to develop familiarity and social bonds.

We use the word ‘partial’ to describe this fusion because it is incomplete due, in part, to a sex difference in mixed group associations. We were much more likely to find male WSR dolphins in mixed groups. The 46 WSR individuals documented off Bimini included males and females in approximately equal numbers; however, there were 13 WSR males and only 3 females that met our generous criteria for COA analyses. The single high COA for female mixed-origin dyads was between two juveniles. By contrast, high mixed-origin COAs involved three Bimini and four WSR males including two strong associations between adult males.

Strong, long-term bonds between two or three males have been described in both Bimini and WSR but may be more common in WSR [20,21]. The function of these associations remains unclear but may involve courtship, based on brief descriptions, but no quantitative studies, in the WSR study (e.g. [55]). The claim of second-order alliances in WSR is based on short-term associations between male pairs and trios [21] but not on aggression against other alliances as occurs among Shark Bay bottlenose dolphins [53]. Instead, such groupings may exist to thwart socio-sexual aggression from male bottlenose dolphins, which is documented in WSR [56,57], though not off Bimini [29,31].

Our finding of significant integration between WSR and Bimini dolphins differs from Herzing *et al.* [22], who concluded that mixed-origin associations were ‘extremely low indicating that WSR dolphins did not integrate into the resident community, but maintained themselves as a distinctly different social cluster’. This statement is supported by their cluster diagram showing the near-complete separation, while ours shows considerable integration. We suggest two explanations for this discrepancy. First, their analysis covered only 3 years and included 2013, the year of arrival; our study includes 5 years and excludes 2013. Fourteen of 79 groups (18%) in their analysis were observed in 2013 compared to 13% of our 197 groups (2013–2018), with a single mixed-origin sighting in 2013 (electronic supplementary material, table S2). Second, our study areas off Bimini may not be concordant, and platforms (day boat versus liveaboard) may allow Herzing *et al.* more time farther from shore to, perhaps, observe more WSR females, which can be deduced from inspection of their social network diagram that includes roughly twice as many WSR individuals as ours. Still, our conclusion that there was significant integration of the two groups is firm, though the majority of WSR dolphins involved were males.

Herzing *et al.* [22] suggested that the WSR dolphins' departure from the Little Bahama Bank was associated with oceanographic shifts that may have influenced prey availability; such changes were not found on the Great Bahama Bank, implying that equivalent changes in prey abundance may not have occurred off Bimini. If dramatic ecological changes drove the WSR dolphins to emigrate to Bimini, it is tempting to compare the spotted dolphin partial fusion to group fusions in primates that occurred under ecologically related demographic stress (e.g. [50,52]). However, neither dolphin community was in decline; indeed, the WSR dolphins were increasing prior to emigration [22]. The cluster of WSR dolphins that moved to Bimini had been observed feeding nocturnally in deep water off the Little Bahama Bank [58]. Though unconfirmed, Bimini dolphins may also forage at night in deep water [20,21]. If spotted dolphins in this region occupy a ‘spinner dolphin niche’ [59], with nocturnal feeding in deep water and daytime predator avoidance in shallows [58,60,61], they may benefit from reduced predation risk in both habitats by forming groups; still, typical group sizes could be maintained without mixing.

Other group formation costs/benefits in this population remain unclear. Both sexes emigrated from WSR in roughly equal numbers. Our OSR estimates do not indicate that males gained access to more reproductively available females by moving. However, once emigrated, mating opportunities may explain why WSR males associated and formed bonds with Bimini females. The formation of bonds between WSR and Bimini males is fascinating in this regard, if such bonds are based on access to females rather than defence of females and calves from aggression by bottlenose dolphins (e.g. [62]).

Karczmarski *et al.* [63] documented a similar immigration event during their study of long-beaked spinner dolphins (*Stenella longirostris longirostris*) in their diurnal resting lagoon on Midway Atoll. An immigrant group of approximately 60 individuals initially rested in an area unused by the residents, who sometimes chased the newcomers. After several months, the immigrant group began using the residents' resting areas but at different times. Nine months after the immigration, the two groups sometimes mingled with affiliative interactions, but separated into their original groups. Further integration after this period was observed (L. Karczmarski, personal communication). The spotted dolphin partial fusion off Bimini occurred in the context of a fission–fusion grouping pattern, with mixed subgroups, whereas the spinner dolphin communities interacted as two large stable groups. Also, the low cost of locomotion and study area size would readily allow two communities off Bimini to completely avoid each other during daytime activities.

Although hostile interactions between groups is typical for mammal social groups, tolerant intergroup interactions have been documented (e.g. [64]). Perhaps the best comparison for spotted dolphins is the bonobo (*P. paniscus*). Bonobos exhibit fission–fusion grouping with female emigration, but females form coalitions with unrelated females to dominate males and each other [65,66]. Unlike the often violent intergroup interactions in chimpanzees, different bonobo communities often mix, sometimes for days, especially when feeding competition is low during high fruit abundance [67,68]. Although aggression increases during intergroup encounters, affiliative interactions are common and intergroup coalitions have been reported [69].

As in bonobos, the associations we documented between these two previously separate spotted dolphin communities included affiliative interactions. Spotted dolphins have a highly dynamic fission–fusion grouping system and we have never observed all community members together in one group. Mixed dolphin community associations always occurred in subgroups, as is typically the case for bonobos, but in unusual cases up to 100 individuals from four different bonobo communities were observed together [67]. Greater participation by male WSR dolphins suggests that mating opportunities may play a role in mixed group formation, as in bonobos [68]. Spotted dolphins’ fission–fusion grouping also renders male defence of females impossible except possibly for short periods, as occurs in some bottlenose dolphins [53], whose non-kin, strategic alliances are largely developed among similar age peers that associated as juveniles [70,71]. The formation of male–male bonds among previously unfamiliar spotted dolphins suggests a similar strategic flexibility. We also appreciate that the benefits of bonds we observe forming during daylight hours in shallow areas may manifest themselves at night in deeper water, during possible cooperative feeding on schooling fish or defence against predators. These are areas for future research.

More broadly, the affiliative interactions and apparent ease of integration between members of two spotted dolphin communities indicate that the adaptations for aggressive responses to non-group members that are so common in social terrestrial mammals are diminished in spotted dolphins, just as in bonobos. We suggest that a similar psychology may be present in many other delphinids for whom resource defence is impractical.

## Data Availability

Data and code used for analyses are available from the Dryad Digital Repository: https://www.doi.org/10.5061/dryad.612jm644s [72].

## References

[1] Wrangham RW. 1980 An ecological model of female-bonded primate groups. Behaviour **75**, 262-300. (10.1163/156853980x00447)

[2] Alba-Mejia L, Caillaud D, Montenegro OL, Sánchez-Palomino P, Crofoot MC. 2013 Spatiotemporal interactions among three neighboring groups of free-ranging white-footed tamarins (*Saguinus leucopus*) in Colombia. Int. J. Primatol. **34**, 1281-1297. (10.1007/s10764-013-9740-6)

[3] Greenwood PJ. 1980 Mating systems, philopatry and dispersal in birds and mammals. Anim. Behav. **28**, 1140-1162. (10.1016/S0003-3472(80)80103-5)

[4] Li YL, Wang L, Wu JW, Ye XP, Garber PA, Yan Y, Liu JH, Li BG, Qi XG. 2020 Bachelor groups in primate multilevel society facilitate gene flow across fragmented habitats. Curr. Zool. **66**, 113-122. (10.1093/cz/zoaa006) 32211037 PMC7083096

[5] Meikle DB, Vessey SH. 1981 Nepotism among rhesus monkey brothers. Nature **294**, 160-116. (10.1038/294160a0) 7197754

[6] Pusey AE, Packer C. 1987 The evolution of sex-biased dispersal in lions. Behaviour **101**, 275-310. (10.1163/156853987X00026)

[7] Williams JM, Oehlert GW, Carlis JV, Pusey AE. 2004 Why do male chimpanzees defend a group range? Anim. Behav. **68**, 523-532. (10.1016/j.anbehav.2003.09.015)

[8] Baird RW. 2000 The killer whale: foraging specializations and group hunting. In Cetacean societies: field studies of dolphins and whales (eds J Mann, RC Connor, PL Tyack, H Whitehead), pp. 127-153. Chicago, IL: The University of Chicago Press.

[9] Connor RC, Wells RS, Mann J, Read AJ. 2000 The bottlenose dolphin: social relationships in a fission-fusion society. In Cetacean societies: field studies of whales and dolphins (eds J Mann, RC Connor, PL Tyack, H Whitehead), pp. 91-126. Chicago, IL: The University of Chicago Press.

[10] Connor R, Mann J, Watson-Capps J. 2006 A sex-specific affiliative contact behavior in Indian Ocean bottlenose dolphins, *Tursiops* sp. Ethology **112**, 631-638. (10.1111/j.1439-0310-2006.01203.x)

[11] Sakai M, Hishii T, Takeda S, Kohshima S. 2006 Flipper rubbing behaviors in wild bottlenose dolphins (*Tursiops aduncus*). Mar. Mamm. Sci. **22**, 966-978. (10.1111/j.1748-7692.2006.00082.x) 16569444

[12] Sakai M, Morisaka T, Kogi K, Hishii T, Kohshima S. 2010 Fine-scale analysis of synchronous breathing in wild Indo-Pacific bottlenose dolphins (*Tursiops aduncus*). Behav. Process. **83**, 48-53. (10.1016/j.beproc.2009.10.001) 19850113

[13] Weiss MN et al. 2021 Age and sex influence social interactions, but not associations, within a killer whale pod. Proc. R. Soc. B **288**, 20210617. (10.1098/rspb.2021.0617) PMC8206696 34130498

[14] Nichols HJ, Arbuckle K, Fullard K, Amos W. 2020 Why don't long-finned pilot whales have a widespread postreproductive lifespan? Insights from genetic data. Behav. Ecol. **31**, 508-518. (10.1093/beheco/arz211)

[15] Tsai YJJ, Mann J. 2013 Dispersal, philopatry, and the role of fission-fusion dynamics in bottlenose dolphins. Mar. Mamm. Sci. **29**, 261-279. (10.1111/j.1748-7692.2011.00559.x)

[16] Connor RC. 2000 Group living in whales and dolphins. In Cetacean societies: field studies of whales and dolphins (eds J Mann, RC Connor, PL Tyack, H Whitehead), pp. 199-218. Chicago, IL: The University of Chicago Press.

[17] Randić S, Connor RC, Sherwin WB, Krützen M. 2012 A novel mammalian social structure in Indo-Pacific bottlenose dolphins (*Tursiops* sp.): complex male alliances in an open social network. Proc. R. Soc. B **279**, 3083-3090. (10.1098/rspb.2012.0264) PMC3385473 22456886

[18] Hunt TN, Allen SJ, Bejder L, Parra GJ. 2019 Assortative interactions revealed in a fission-fusion society of Australian humpback dolphins. Behav. Ecol. **30**, 914-927. (10.1093/beheco/arz029)

[19] Bertulli CG, Rasmussen MH, Rosso M. 2021 Fission-fusion dynamics of a pelagic delphinid in the arctic: the white-beaked dolphin (*Lagenorhynchus albirostris*). Int. Zoo. **16**, 512-526. (10.1111/1749-4877.12524) 33559948

[20] Danaher-Garcia NA, Melillo-Sweeting K, Dudzinski KM. 2020 Social structure of Atlantic spotted dolphins (*Stenella frontalis*) off Bimini, The Bahamas (2003–2016): alternate reasons for preferential association in delphinids. Acta Ethol. **23**, 9-21. (10.1007/s10211-019-00329-3)

[21] Elliser CR, Herzing DL. 2014 Long-term social structure of a resident community of Atlantic spotted dolphins, *Stenella frontalis*, in the Bahamas 1991–2002. Mar. Mamm. Sci. **30**, 308-328. (10.1111/mms.12039)

[22] Herzing DL, Augliere BN, Elliser CR, Green ML, Pack AA. 2017 Exodus! Large-scale displacement and social adjustments of resident Atlantic spotted dolphins (*Stenella frontalis*) in the Bahamas. PLoS ONE **12**, e0180304. (10.1371/journal.pone.0180304) 28792947 PMC5549894

[23] Elliser CR, Herzing DL. 2012 Community structure and cluster definition of Atlantic spotted dolphins, *Stenella frontalis*, in the Bahamas. Mar. Mamm. Sci. **28**, E486-E502. (10.1111/j.1748-7692.2012.00576.x)

[24] Kaplan JD, Connor RC. 2007 A preliminary examination of sex differences in tactile interactions among juvenile Atlantic spotted dolphins (*Stenella frontalis*). Mar. Mamm. Sci. **23**, 943-953. (10.1111/j.1748-7692.2007.00142.x)

[25] Paolos RD, Dudzinski KM, Kuczaj SA. 2008 The role of touch in select social interactions of Atlantic spotted dolphin (*Stenella frontalis*) and Indo-Pacific bottlenose dolphin (*Tursiops aduncus*). J. Ethol. **26**, 153-164. (10.1007/s10164-007-0047-y)

[26] Dudzinski KM, Gregg JD, Paulos RD, Kuczaj SA. 2010 A comparison of pectoral fin contact behaviour for three distinct dolphin populations. Behav. Process. **84**, 559-567. (10.1016/j.beproc.2010.02.013) 20176094

[27] Dudzinski KM, Gregg J, Melillo-Sweeting K, Seay B, Levengood A, Kuczaj SA. 2012 Tactile contact exchanges between dolphins: self-rubbing versus inter-individual contact in three species from three geographics. Int. J. Comp. Psych. **25**, 21-43.

[28] Dudzinski KM, Danaher-Garcia N, Gregg JD. 2013 Pectoral fin contact between dolphin dyads at Zoo Duisburg, with comparison to other dolphin study populations. Aquat. Mamm. **39**, 335-343. (10.1578/AM.39.4.2013.335)

[29] Eierman LE, Laccetti K, Melillo-Sweeting K, Kaplan JD. 2019 Interspecies pectoral fin contact between bottlenose dolphins and Atlantic spotted dolphins off Bimini, The Bahamas. Anim. Behav. **157**, 167-176. (10.1016/j.anbehav.2019.09.002)

[30] Elliser CR, Herzing DL. 2014 Social structure of Atlantic spotted dolphins, *Stenella frontalis*, following environmental disturbance and demographic changes. Mar. Mamm. Sci. **30**, 329-347. (10.1111/mms.12038)

[31] Melillo KE, Dudzinski KM, Cornick LA. 2009 Interactions between Atlantic spotted (*Stenella frontalis*) and bottlenose (*Tursiops truncatus*) dolphins off Bimini, The Bahamas, 2003–2007. Aquat. Mamm. **35**, 281-291. (10.1578/AM.35.2.2009.281)

[32] Perrin WF. 1970 Color pattern of the eastern Pacific spotted porpoise *Stenella graffmani* Lönnberg (Cetacean, Delphinidae). Zoologica **54**, 135-149. (10.5962/p.203253)

[33] Herzing DL. 1997 The life history of free-ranging Atlantic spotted dolphins (*Stenella frontalis*): age classes, color phases, and female reproduction. Mar. Mamm. Sci. **13**, 576-595. (10.1111/j.1748-7692.tb00085.x)

[34] Dudzinski K. 1998 Contact behavior and signal exchange in Atlantic spotted dolphins (*Stenella frontalis*). Aquat. Mamm. **24**, 129-142.

[35] Dudzinski KM, Clark CW, Würsig B. 1995 A mobile video-acoustic array for simultaneously recording dolphin behavior and vocalizations underwater. Aquat. Mamm. **21**, 187-193.

[36] Dudzinski KM, Gregg JD, Ribic CA, Kuczaj SA. 2009 A comparison of pectoral fin contact between two different wild dolphin populations. Behav. Process. **80**, 182-190. (10.1016/j.beproc.2008.11.011) 19070654

[37] Altmann J. 1974 Observational study of behavior: sampling methods. Behaviour **49**, 227-267. (10.1163/156853974X00534) 4597405

[38] Mann J. 1999 Behavioral sampling methods for cetaceans: a review and critique. Mar. Mamm. Sci. **15**, 102-122. (10.1111/j.1748-7692.1999.tb00784.x)

[39] Dudzinski KM. 1996 Communication and behavior in the Atlantic spotted dolphin (*Stenella frontalis*): relationships between vocal and behavioral activities. Dissertation, Texas A&M University.

[40] Themelin M, Ribic CA, Melillo-Sweeting K, Dudzinski KM. 2020 New approach for relationships quality study in dolphins. Behav. Process. **181**, 104260. (10.1016/j.beproc.2020.104260) 33017667

[41] R Core Team. 2018 R: a language and environment for statistical computing. Vienna, Austria: R Foundation for Statistical Computing. https://www.R-project.org/.

[42] Fox J, Weisberg S. 2019 An {R} companion to applied regression, 3rd edn. Thousand Oaks, CA: Sage. See https://socialsciences.mcmaster.ca/jfox/Books/Companion/.

[43] Hothorn T, Bretz F, Westfall P. 2008 Simultaneous inference in general parametric models. Biom. J. **50**, 346-363. (10.1002/bimj.200810425) 18481363

[44] Whitehead H. 2009 SOCPROG programs: analyzing animal social structures. Behav. Ecol. Sociobiol. **63**, 765-778. (10.1007/s00265-008-0697-y)

[45] Cairns SJ, Schwager SJ. 1987 A comparison of association indices. Anim. Behav. **35**, 1454-1469. (10.1016/S0003-3472(87)80018-0)

[46] Whitehead H. 2008 Analyzing animal societies: quantitative methods for vertebrate social analysis. Chicago, IL: The University of Chicago Press.

[47] Borgatti SP, Everett MG, Freeman LC. 2002 Ucinet 6 for Windows: software for social network analysis. Harvard, MA: Analytic Technologies.

[48] Newman MEJ. 2004 Analysis of weighted networks. Phys. Rev. E **70**, 056131. (10.1103/PhysRevE.70.056131) 15600716

[49] Emlen ST. 1976 Lek organization and mating strategies in the bullfrog. Behav. Ecol. Sociobiol. **1**, 283-313. (10.1007/bf00300069)

[50] Isbell LA, Cheney DL, Seyfarth RM. 1991 Group fusions and minimum group sizes in vervet monkeys (*Cercopithecus aethiops)*. Am. J. Primatol. **25**, 57-65. (10.1002/ajp.1350250106) 31952380

[51] Isbell LA. 1990 Sudden short-term increase in mortality of vervet monkeys (*Cercopithecus aethiops*) due to leopard predation in Amboseli National Park, Kenya. Am. J. Primatol. **40**, 41-52. (10.1002/ajp.1350210105) 31963987

[52] Takahata Y, Suzuki S, Okayasu N, Hill D. 1994 Troop extinction and fusion in wild Japanese macaques of Yakushima Island, Japan. Am. J. Primatol. **33**, 317-322. (10.1002/ajp.1350030406) 31936942

[53] Connor RC, Krützen M. 2015 Male dolphin alliances in Shark Bay: changing perspectives in a 30-year study. Anim. Behav. **103**, 223-235. (10.1016/j.anbehav.2015.02.019)

[54] Frère CH, Krützen M, Mann J, Connor RC, Bejder L, Sherwin WB. 2010 Social and genetic interactions drive fitness variation in a free-living dolphin population. Proc. Natl Acad. Sci. USA **107**, 19 949-19 954. (10.1073/pnas.1007997107) PMC2993384 21041638

[55] Herzing D. 1996 Vocalizations and associated underwater behavior of free-ranging Atlantic spotted dolphins, *Stenella frontalis* and bottlenose dolphins, *Tursiops truncatus*. Aquat. Mamm. **22**, 61-79. (10.12966/abc.02.02.2015)

[56] Elliser CR, Herzing DL. 2016 Changes in interspecies association patterns of Atlantic bottlenose dolphins, *Tursiops truncatus*, and Atlantic spotted dolphins, *Stenella frontalis*, after demographic changes related to environmental disturbance. Mar. Mamm. Sci. **32**, 602-618. (10.1111/mms.12289)

[57] Myers AJ, Herzing DL, Bjorklund DF. 2017 Synchrony during aggression in adult male Atlantic spotted dolphins (*Stenella frontalis*). Acta Ethol. **20**, 175-185. (10.1007/s10211-017-0262-7)

[58] Herzing DL, Elliser CR. 2014 Nocturnal feeding of Atlantic spotted dolphins (*Stenella frontalis*) in the Bahamas. Mar. Mamm. Sci. **30**, 367-373. (10.1111/mms.12016)

[59] Norris KS, Dohl TP. 1980 Behavior of the Hawaiian spinner dolphin, *Stenella longirostris*. Fish. Bull. **77**, 821-849.

[60] Melo CLC, Santos RA, Bassoi M, Araújo AC, Lailson-Brito J, Dorneles PR, Azevedo AF. 2010 Feeding habits of delphinids (Mammalia: Cetacean) from Rio de Janeiro State, Brazil. J. Mar. Biol. **90**, 1509-1515. (10.1017/S0025315409991639)

[61] Fernandez R, Santos MB, Carrillo M, Tejedor M, Pierce GJ. 2009 Stomach contents of cetaceans stranded in the Canary Islands 1996–2006. J. Mar. Biol. **89**, 873-883. (10.1017/S0025315409000290)

[62] Cusick JA, Herzing DL. 2014 The dynamic of aggression: how individual and group factors affect the long-term interspecific aggression between two sympatric species of dolphin. Ethology **120**, 287-303. (10.1111/eth.12204)

[63] Karczmarski L, Würsig B, Gailey G, Larson KW, Vanderlip C. 2005 Spinner dolphins in a remote Hawaiian atoll: social grouping and population structure. Behav. Ecol. **16**, 675-685. (10.1093/beheco/ari028)

[64] Furuichi T. 2020 Variation in intergroup relationships among species and among and within local populations of African apes. Int. J. Primatol. **41**, 203-223. (10.1007/s10764-020-00134-x)

[65] Kano T. 1982 The social group of pygmy chimpanzees (*Pan paniscus*) of Wamba. Primates **23**, 171-188. (10.1007/BF02381159)

[66] Tokuyama N, Furuichi T. 2016 Do friends help each other? Patterns of female coalition formation in wild bonobos at Wamba. Anim. Behav. **119**, 27-35. (10.1016/j.anbehav.2016.06.021)

[67] Sakamaki T, Ryu H, Toda K, Tokuyama N, Furuichi T. 2018 Increased frequency of intergroup encounters in wild bonobos (*Pan paniscus*) around the yearly peak in fruit abundance at Wamba. Int. J. Primatol. **39**, 685-704. (10.1007/s10764-018-0058-2)

[68] Lucchesi S, Cheng L, Janmaat KRL, Mundry R, Pisor A, Surbeck M. 2020 Beyond the group: how food, mates, and group size influence intergroup encounters in wild bonobos. Behav. Ecol. **31**, 519-532. (10.1093/beheco/arz214)

[69] Tokuyama N, Sakamaki T, Furuichi T. 2019 Inter-group aggressive interaction patterns indicate male mate defense and female cooperation across bonobo groups at Wamba, Democratic Republic of the Congo. Am. J. Phys. Anthropol. **170**, 535-550. (10.1002/ajpa.23929) 31724741

[70] Gerber L et al. 2020 Affiliation history and age similarity predict alliance formation in adult male bottlenose dolphins. Behav. Ecol. **31**, 361-370. (10.1093/beheco/arz195) 32210525 PMC7083095

[71] Gerber L et al. 2021 Cooperative partner choice in multi-level male dolphin alliances. Sci. Rep. **11**, 6901. (10.1038/s41598-021-85583-x) 33767258 PMC7994371

[72] Danaher-Garcia N, Melillo-Sweeting K. 2022 Sighting data and tactile events logged from mixed-group underwater interactions. Dryad Digital Repository. (10.5061/dryad.612jm644s)

